# Leveraging 3D Molecular Spatial Visual Information and Multi‐Perspective Representations for Drug Discovery

**DOI:** 10.1002/advs.202512453

**Published:** 2025-10-15

**Authors:** Zimai Zhang, Xi Zhou, Yujie Qi, Xiaobo Zhu, Xun Deng, Feng Tan, Yuan Huang, Lun Hu, Zhuhong You, Pengwei Hu

**Affiliations:** ^1^ Xinjiang Technical Institute of Physics and Chemistry Chinese Academy of Sciences Urumqi 830011 China; ^2^ School of Software Xinjiang University Urumqi 830046 China; ^3^ School of Computer Science and Technology University of Chinese Academy of Sciences Beijing 100049 China; ^4^ Al and Quantum Lab Merck KGaA 64293 Darmstadt Germany; ^5^ School of Computer Science Northwestern Polytechnical University Xian 710129 China

**Keywords:** drug 3D molecular spatial visual information, drug discovery, multi‐perspective learning

## Abstract

Drug discovery remains a costly and time‐intensive process, where accurate identification of drug associations is critical for therapeutic development. Existing computational approaches predominantly rely on sequence‐derived or 2D molecular representations, often overlooking the intrinsic 3D complexity of small molecules. Here, a deep learning framework is presented that directly learns from 3D molecular spatial visual information, capturing geometric, topological, and stereochemical features from spatial renderings. By integrating this spatial information with traditional molecular descriptors, unified multi‐perspective representations are constructed that better reflect molecular structure and function. Across benchmark tasks involving drug–microRNA, drug–drug, and drug–protein interaction prediction, this model consistently outperforms conventional fingerprint‐based baselines. Interpretability analyses show that the model attends to biologically relevant substructures, highlighting the value of 3D molecular spatial visual information in molecular recognition. These findings demonstrate the potential of spatially informed learning to enhance predictive performance and provide mechanistic insights in computational drug discovery.

## Graphical abstract

A deep learning framework called MolVisGNN is proposed to fuse 3D molecular visual information of drugs with multi‐source features, which proves the importance of 3D molecular visual information of drugs and the advancedness of this model in the field of drug discovery, and provides a reference for how to more comprehensively express small molecule drugs in deep learning in the future.

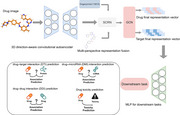

## 1 Introduction

Drug discovery is an inherently complex and time‐intensive process, yet it remains foundational to biomedical innovation. Traditionally, the identification of therapeutic candidates has relied on laborious and costly biological experiments, which are often constrained by environmental variability such as temperature and humidity.^[^ ^1^ ^]^ With the emergence of artificial intelligence (AI), the drug development landscape is undergoing a paradigm shift. Recent advances in machine learning and deep learning have enabled large‐scale virtual screening, providing more efficient and scalable alternatives to conventional wet‐lab methods.^[^ ^2^, ^3^, ^4^ ^]^

Currently, many deep learning‐related technologies have been applied to related fields of drug discovery.^[^ ^5^, ^6^, ^7^ ^]^ In contrast to experimental pipelines, AI‐based models encode drug‐related information into standardized feature representations, enabling them to capture complex biochemical patterns and relationships. A critical component of these models is the construction of informative drug embeddings. Most existing frameworks derive such embeddings from 1D (e.g., SMILES strings or sequence similarities) or 2D (e.g., molecular graphs or topological fingerprints) data.^[^ ^8^ ^]^ However, these representations often neglect the intrinsic 3D characteristics of drug molecules, which are essential for capturing conformational and spatial features that govern molecular recognition and binding.

In biological systems, drug molecules interact with their targets within 3D space. Accordingly, many structure‐based computational approaches–such as molecular docking, molecular dynamics simulations, and structure‐guided pharmacology–already incorporate 3D structural information to model molecular interactions.^[^ ^9^ ^]^ Despite this, relatively few deep learning frameworks have fully embraced 3D structure as an integral component of drug representation. Recent studies have begun addressing this limitation by introducing 3D‐aware molecular graphs or distance matrices.^[^ ^10^, ^11^, ^12^ ^]^ While these approaches improve upon earlier models, they often abstract away finer geometric or physicochemical properties, such as curvature or spatial orientation of key substructures.

Complementing these efforts, recent research has explored image‐based molecular encodings. For instance, ImageMol^[^ ^13^ ^]^ leveraged 2D molecular renderings to extract drug features, while VideoMol^[^ ^14^ ^]^ utilized 3D molecular videos with large‐scale pretraining. Inspired by these advances, we propose a novel framework to visually encode the 3D structural information of drug molecules and learn multi‐perspective representations. Rather than manually designing spatial descriptors, we employ scientific visualization software to render ball‐and‐stick representations that preserve spatial conformation, bonding patterns, and stereochemical features. These 3D‐aware images, representing 3D molecular spatial visual information, are then processed by a deep neural encoder and fused with conventional drug features to form a multi‐view embedding that integrates both structural and semantic contexts.

We evaluate our model across multiple interaction prediction tasks, including drug–microRNA, drug–drug, and drug–protein associations, as well as toxicity prediction on the LD_50_ dataset. To address the diverse biological context underlying these tasks, our framework combines biologically informed feature initialization with task‐specific training. Specifically, a multimodal drug encoder integrates 1D, 2D, and 3D features into a unified representation, while proteins and miRNAs are processed using dedicated encoders to preserve domain‐specific information. Furthermore, while the backbone architecture is shared, each task is trained independently, avoiding negative transfer and allowing the model to fine‐tune its representation in a task‐adaptive manner. In all cases, integrating 3D molecular spatial visual information leads to consistent improvements over traditional fingerprint‐based baselines. To further validate robustness, we conducted a series of evaluations, including ablation studies, scaffold‐based splitting, orphan‐target tests, and diverse case analyses. Interpretability experiments additionally show that the model attends to functionally relevant substructures, indicating that even simplified 3D visual representations can capture biologically meaningful spatial cues. Collectively, these findings highlight the potential of deep learning frameworks leveraging 3D molecular spatial visual information to improve the accuracy, interpretability, and generalizability of structure‐informed virtual drug discovery.

## 2 Results

### 2.1 Overview of the MolVisGNN Architecture

**Figure 1:**
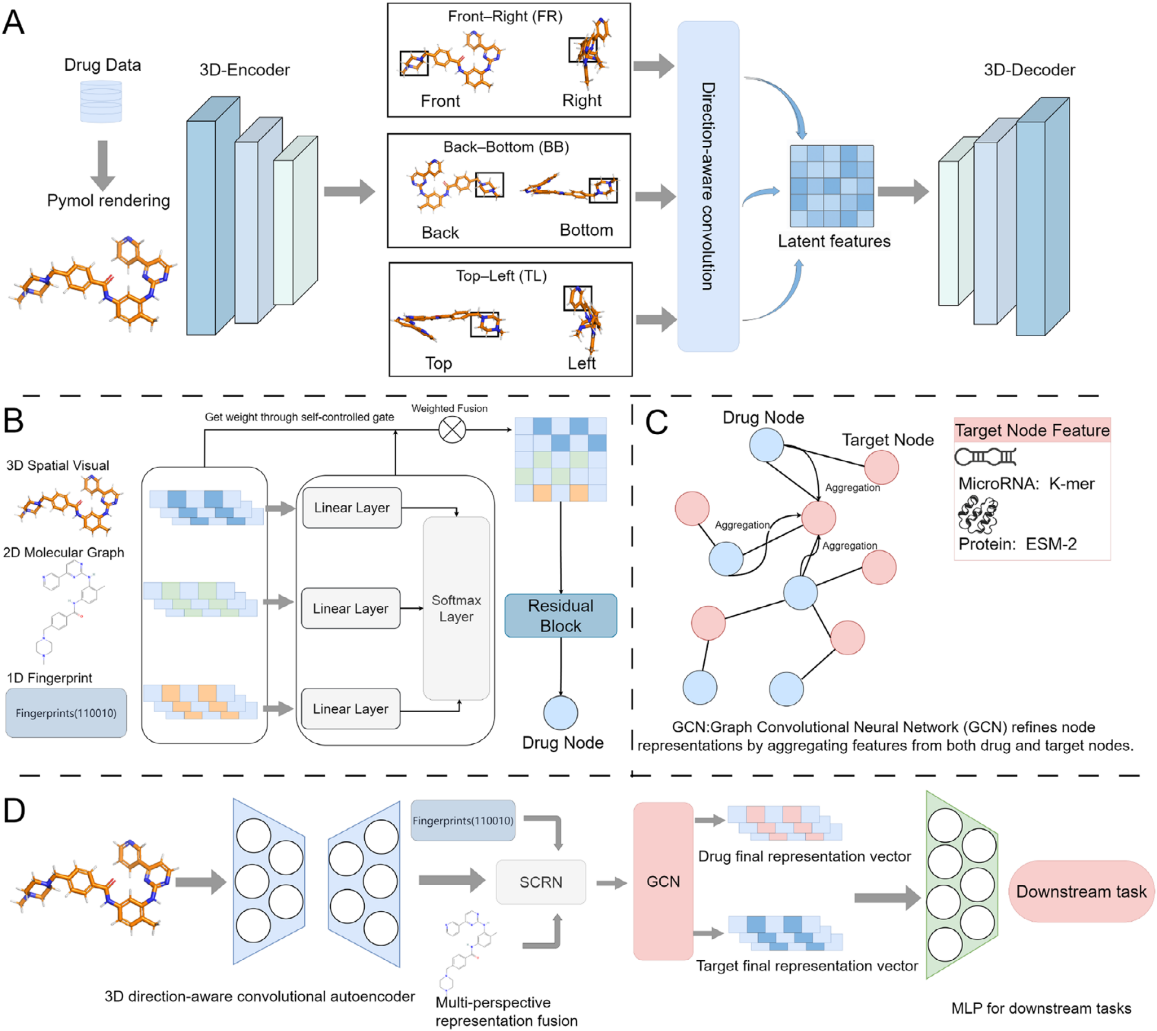
Overview of the MolVisGNN framework for drug discovery. A) Six orthogonal views of PyMOL renderings are passed through a 3D direction‐aware convolutional autoencoder to extract global and local expressions. B) We propose a Self‐Controlled Gated Residual Network (SCRN) that linearly projects 3D spatial, 2D graph, and 1D fingerprint features into a shared latent space, applies a gating mechanism to compute attention weights for fusion, and refines the fused vector through a 1D residual convolutional block to produce the final drug embedding. C) A graph convolutional network (GCN) is used to propagate and refine drug and target features over an interaction graph. Targets include proteins (encoded by ESM‐2) and microRNAs (encoded by k‐mer). D) End‐to‐end inference workflow of MolVisGNN.

As illustrated in **Figure** **1**, the architecture of MolVisGNN comprises three core components: extraction of 3D molecular spatial visual information, fusion of multi‐perspective molecular representations, and graph‐based relational reasoning for downstream prediction tasks.

Before training the model, the molecular conformations were optimized using the MMFF94s force field to minimize energy, yielding 3D molecular structures with more reasonable geometries, lower energies, and closer approximations to their real physical states. Six orthogonal 3D views–top, bottom, left, right, front, and back–were then rendered using PyMOL.^[^ ^15^ ^]^ These views preserve key spatial information, such as bond angles, stereochemistry, and conformational geometry. The multi‐view images were stored in .npy format and fed into a 3D direction‐aware convolutional autoencoder to generate global structural embeddings that capture the overall molecular scaffold and atomic bonding arrangements.

To capture local spatial asymmetry and directional features, which often play a decisive role in distinguishing structural isomers, we use a Direction‐aware convolution mechanism. Specifically, six 3D views are grouped into three pairs of semantically meaningful images ‐ front right (FR), back bottom (BB), and top left (TL), and processed by independent 3D convolutional layers. This architecture, implemented in the form of DirectionalConvBlock, enables the model to learn view‐specific spatial features to highlight pharmacologically relevant substructures such as aromatic rings, heteroatoms, and functional motifs. By learning filters in fixed directions, the model improves the ability to distinguish stereoisomers and structural isomers, which often exhibit similar overall frameworks but differ in functional behaviors.

In parallel, MolVisGNN also incorporates conventional molecular descriptors: 1D molecular fingerprints encoding substructure presence and 2D graph‐based features capturing atom connectivity and bond topology. These heterogeneous features are adaptively fused via a self‐controlled gate residual network (SCRN), which dynamically balances modality contributions to construct an integrated representation encompassing spatial, topological, and substructural signals.

Finally, a graph convolutional network (GCN)^[^ ^16^ ^]^ performs relational reasoning over a heterogeneous drug–biomolecule interaction graph. Drugs are represented by fused embeddings, while targets (e.g., proteins and microRNAs) are encoded using appropriate biological embeddings (e.g., ESM‐2 for proteins and k‐mer embeddings for miRNAs). Through message passing, the GCN aggregates feature and topology information to support downstream prediction tasks, including drug–target interaction (DTI), drug–drug interaction (DDI), and drug–microRNA interaction (DMI).

In summary, MolVisGNN offers a unified, multimodal framework that integrates directional spatial encoding with relational learning to capture the complex structural and interaction‐specific features underlying pharmacological behavior.

### 2.2 Evaluation Metrics and Tasks

Our goal is to develop a generalizable framework that can be broadly applied to diverse drug discovery tasks. In practice, computational drug discovery encompasses a variety of predictive objectives, each targeting different molecular associations and biological contexts. To assess the applicability and robustness of our proposed approach, we evaluated it on three representative and biologically meaningful tasks: drug–target interaction (DTI) prediction, drug–drug interaction (DDI) prediction, and drug–microRNA (DMI) interaction prediction, as well as toxicity prediction on the LD_50_ dataset. As an emerging front in computational pharmacology, DMI prediction has recently attracted increasing attention due to the regulatory role of microRNAs in disease mechanisms and drug response.

For evaluation, we employed several standard metrics, including the area under the receiver operating characteristic curve (ROC‐AUC), the area under the precision–recall curve (AUPR), precision (Pre), recall, and the F1‐score. During training, unknown associations were treated as negative samples, with a 1:1 ratio maintained between positive and negative instances to ensure balanced learning. For the toxicity prediction task, we adopted Root Mean Squared Error (RMSE), Mean Absolute Error (MAE), Median Absolute Error (MedAE), Mean Absolute Percentage Error (MAPE), the Coefficient of Determination (R^2^), and the Explained Variance Score (EVS) as evaluation metrics.

### 2.3 Benchmarking 3D Molecular Visual Information Against Molecular Fingerprints

To preliminarily evaluate the effectiveness of 3D molecular spatial visual information, we conducted comparative experiments using widely adopted molecular fingerprint representations. Molecular fingerprints encode chemical structure into fixed‐length binary or numerical vectors, where each bit indicates the presence or absence of a specific substructure or functional group. These representations are commonly used in cheminformatics due to their computational efficiency and interpretability.

We selected three representative fingerprint types as baselines: 1) Topological Torsion fingerprints,^[^ ^17^ ^]^ which encode atom sequences and their torsional relationships within four‐membered substructures, capturing stereochemistry and local topology; 2) Morgan fingerprints (Extended‐Connectivity Fingerprints),^[^ ^18^ ^]^ which iteratively hash local atomic neighborhoods to capture molecular topology and chemical environments; and 3) MACCS keys,^[^ ^19^ ^]^ a fixed set of structural fragments and functional groups commonly used in drug screening tasks. These fingerprinting methods serve as robust baselines for virtual screening and molecular similarity modeling.

**Table** **1** presents the comparative results of these feature representations across three tasks: drug–target interaction (DTI), drug–drug interaction (DDI), and drug–microRNA interaction (DMI) prediction. In the DTI task, the Morgan fingerprint slightly outperforms the only‐3D model–where only 3D molecular spatial visual information is used–achieving the highest ROC‐AUC (0.9596) and F1‐score (0.9167). This is likely due to the fingerprint's capacity to encode chemical substructures critical for target binding. In contrast, for the DDI task, the only‐3D approach achieves the best performance, with a ROC‐AUC of 0.9763 and an F1‐score of 0.9546, indicating the strength of 3D spatial representations in modeling structural interactions between drug compounds. The largest performance gain from 3D information is observed in the DMI task, where the only‐3D configuration achieves the highest ROC‐AUC (0.9826), AUPR (0.9823), and F1‐score (0.9295), significantly outperforming all fingerprint‐based baselines. These results highlight the discriminative power of 3D structural features in capturing the spatial and physicochemical context of drug–microRNA associations.

**Table 1:** Performance comparison of 3D molecular spatial visual information against traditional fingerprint‐based methods across DTI, DDI, and DMI tasks.

Method	ROC‐AUC	AUPR	Accuracy	Precision	F1‐score
**DTI**					
only‐3D	0.9496	0.9283	0.9005	0.8440	0.9081
**Morgan**	**0.9596**	**0.9503**	**0.9109**	**0.8604**	**0.9167**
MACC	0.9551	0.9492	0.8873	0.8328	0.8963
TopologicalTorsion	0.9350	0.9220	0.8791	0.8389	0.8867
**DDI**					
**only‐3D**	**0.9763**	**0.9603**	**0.9552**	**0.9300**	**0.9546**
Morgan	0.8695	0.7675	0.8300	0.8061	0.8363
MACC	0.9206	0.8860	0.8595	0.8312	0.8653
TopologicalTorsion	0.8994	0.7958	0.8920	0.8418	0.8994
**DMI**					
**only‐3D**	**0.9826**	**0.9823**	**0.9821**	**0.9122**	**0.9295**
Morgan	0.9367	0.9197	0.8643	0.8384	0.8693
MACC	0.9572	0.9519	0.8936	0.8722	0.8965
TopologicalTorsion	0.9315	0.9207	0.8431	0.8741	0.8363

The superior performance of Morgan fingerprints in the DTI task can be attributed to their ability to encode localized substructures such as amino, carboxyl, or aromatic groups–functional moieties that often mediate specific interactions with protein residues like lysine or glutamate. By adjusting the fingerprint radius, Morgan representations can flexibly capture both fine‐grained and broader molecular contexts, making them particularly effective for modeling drug–target interactions. Overall, our findings suggest that while 3D structural features offer notable advantages for DDI and DMI prediction, combining them with 2D fingerprint‐based features may further enhance DTI prediction performance.

### 2.4 Multi‐Perspective Representations Enhances the Expressive Power of Drugs

Although 3D molecular spatial visual information offers valuable insights into spatial conformation, our experimental results indicate that it alone is insufficient to fully capture the structural and functional complexity required for accurate drug–target interaction prediction. In particular, essential chemical characteristics–such as electronic properties, functional groups, and atomic connectivity–are only partially conveyed through visual 3D representations.

To address these limitations, we designed a multi‐representation fusion strategy that integrates 3D molecular spatial visual information with two complementary molecular descriptors: molecular fingerprints and graph‐based structural encodings. In this framework, molecular fingerprints serve as 1D descriptors, encoding the presence or absence of substructures and functional moieties in high‐dimensional binary form. Simultaneously, graph‐based molecular representations–extracted using a graph convolutional network (GCN)–^[^ ^20^ ^]^ capture 2D topological features such as atomic connectivity and neighborhood context.

To effectively unify these heterogeneous modalities, we introduce a self‐controlled gated residual network (SCRN). The gating mechanism in SCRN dynamically assigns weights to each modality based on its predictive relevance, while the residual convolutional pathway preserves critical information from each representation. This design allows SCRN to learn complementary features across modalities while maintaining representation integrity and enhancing biological signal integration.

**Figure 2:**
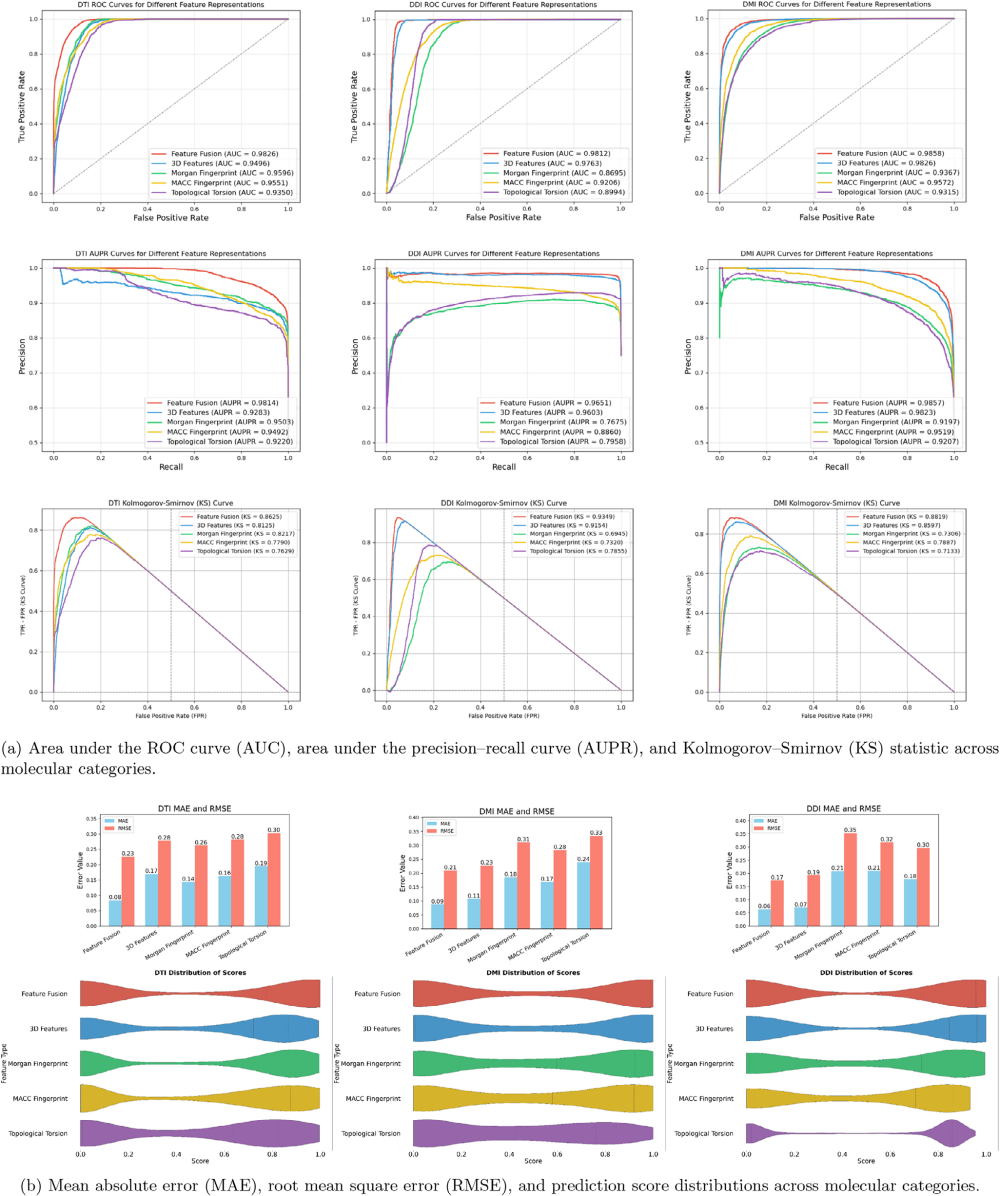
Performance evaluation of the proposed feature fusion model incorporating 3D molecular spatial visual information, compared against three molecular fingerprint baselines.

This fusion strategy results in significant performance improvements, as shown in **Table** **2**. Compared with models that rely exclusively on 3D molecular spatial visual information, the integration of fingerprint‐based and graph‐derived features consistently yields higher predictive accuracy across multiple benchmark tasks. We attribute these gains to the complementary strengths of the three input modalities: 3D molecular spatial visual features capture geometric conformation, fingerprints encode chemical substructures, and molecular graphs represent topological and contextual relationships. Together, these representations enable a more comprehensive and discriminative molecular encoding. As further illustrated in **Figure** **2**a,b, the proposed fusion framework demonstrates robust and consistent performance across diverse evaluation settings.

**Table 2:** Performance comparison of the proposed method with current advanced models across DTI, DDI, and DMI prediction tasks.

Method	ROC‐AUC	AUPR	Accuracy	Precision	F1‐score
**DTI**					
**MolVisGNN**	**0.9761**	**0.9839**	**0.9660**	**0.9634**	**0.9661**
VideoMol	0.9428	0.9378	0.8745	0.8248	0.8835
EviDTI	0.9669	0.9620	0.9436	0.9396	0.9436
DrugBAN	0.9729	0.9685	0.9297	0.9382	0.9291
BINDTI	0.9524	0.9562	0.9007	0.9083	0.8998
CoaDTI	0.8900	0.8333	0.8712	0.8306	0.8799
GAM‐MDR	0.8888	0.8901	0.8700	0.9102	0.8632
**DDI**					
**MolVisGNN**	**0.9812**	**0.9651**	**0.9645**	**0.9560**	**0.9648**
VideoMol	0.8489	0.8378	0.7650	0.7283	0.7825
HTCL‐DDI	0.7456	0.7080	0.6801	0.6598	0.6991
DSN‐DDI	0.9564	0.9504	0.8981	0.8852	0.8998
SSI‐DDI	0.8834	0.8578	0.8085	0.7982	0.8257
**DMI**					
**MolVisGNN**	**0.9858**	**0.9857**	**0.9393**	0.9245	**0.9403**
VideoMol	0.9109	0.9005	0.8388	0.8158	0.8444
HGNNLDA	0.8397	0.8535	0.7184	0.6737	0.7547
AGCLNDA	0.9000	0.9142	0.8362	0.8476	0.8334
GAM‐MDR	0.9696	0.9777	0.8861	**0.9611**	0.8735

In the drug–target interaction (DTI) prediction task, the multi‐perspective model achieves an AUC of 0.9826, reaffirming its superior predictive capacity. The 3D representation model follows with an AUC of 0.9496, while Morgan, MACCS, and Topological Torsion fingerprints achieve AUCs of 0.9596, 0.9551, and 0.9350, respectively, indicating a performance gap in capturing complex interaction features.

A similar trend is observed in AUPR analysis: the multi‐perspective and 3D models reach AUPRs of 0.9814 and 0.9283, respectively, outperforming Morgan (0.9503), MACCS (0.9492), and Topological Torsion (0.9220). KS test results also support these findings, with the multi‐perspective model achieving the highest KS value of 0.8625, followed by the 3D model (0.8125). The fingerprint‐based models perform less favorably, with KS values of 0.8217 (Morgan), 0.7790 (MACCS), and 0.7629 (Topological Torsion), reflecting their reduced distributional discriminability.

In the drug–drug interaction (DDI) task, the multi‐perspective model attains an AUC of 0.9812, confirming the benefit of multimodal integration. The 3D representation model achieves 0.9763, while Morgan, MACCS, and Topological Torsion lag behind with AUCs of 0.8695, 0.9206, and 0.8944, respectively. AUPR results follow a consistent pattern, with the multi‐perspective and 3D models achieving 0.9651 and 0.9603, respectively, outperforming Morgan (0.7675), MACCS (0.8860), and Topological Torsion (0.7958). In terms of KS scores, the multi‐perspective model achieves 0.9349 and the 3D model reaches 0.9154, whereas the fingerprint models yield lower scores: 0.6945 (Morgan), 0.7320 (MACCS), and 0.7855 (Topological Torsion), again confirming the inferior separation capacity of traditional descriptors.

In the drug–microRNA interaction (DMI) prediction task, receiver operating characteristic (ROC) analysis shows that the multi‐perspective model achieves an AUC of 0.9858, demonstrating a strong ability to distinguish between positive and negative samples. The 3D representation model performs comparably with an AUC of 0.9826, highlighting the discriminative value of spatial molecular features. Fingerprint‐based models achieve lower AUCs: 0.9367 (Morgan), 0.9572 (MACCS), and 0.9315 (Topological Torsion), reflecting their limited ability to capture interaction‐specific features. In precision–recall analysis, the multi‐perspective and 3D models reach AUPRs of 0.9857 and 0.9823, respectively, while Morgan, MACCS, and Topological Torsion score 0.9197, 0.9096, and 0.9207. The KS test further reinforces these results: the multi‐perspective model attains a KS value of 0.8819, followed by the 3D model (0.8597), with fingerprint‐based methods performing less effectively–0.7306 (Morgan), 0.7887 (MACCS), and 0.7133 (Topological Torsion).

Across the DTI, DDI, and DMI prediction tasks, the multi‐perspective model consistently outperforms single‐modality approaches across all evaluation metrics, including AUC, AUPR, and KS. These results highlight the advantage of integrating 1D molecular fingerprints, 2D graph‐based structural representations, and 3D molecular spatial visual information. In contrast, traditional fingerprint descriptors such as Morgan, MACCS, and Topological Torsion exhibit limited predictive performance, underscoring the importance of multimodal representation learning for capturing the structural, spatial, and functional complexity of molecular interactions.

### 2.5 Comparison with Other Methods

To ensure a fair and comprehensive evaluation of our model's effectiveness across diverse drug‐related prediction tasks, we selected a range of state‐of‐the‐art baselines tailored to each task domain. For the drug–miRNA interaction (DMI) prediction task, we compared our model against HGNNLDA,^[^ ^21^ ^]^ AGCLNDA,^[^ ^22^ ^]^ and GAM‐MDR,^[^ ^23^ ^]^ which represent recent advances in graph neural networks and attention mechanisms for miRNA association prediction. For drug–drug interaction (DDI) prediction, we adopted HTCL‐DDI,^[^ ^24^ ^]^ DSN‐DDI,^[^ ^25^ ^]^ and SSI‐DDI^[^ ^26^ ^]^ as comparison models, each of which leverages different structural or semantic strategies to capture drug–drug associations. For drug–target interaction (DTI) prediction, we conducted comparative analyses with DrugBAN,^[^ ^27^ ^]^ BINDTI,^[^ ^28^ ^]^ CoaDTI,^[^ ^29^ ^]^ GAM‐MDR,^[^ ^23^ ^]^ and EviDTI,^[^ ^30^ ^]^ which cover attention‐based networks, co‐attention learning frameworks, interpretable architectures, and the latest evidential deep learning (EDL)‐based models. In addition, to assess the efficacy of 3D image‐based representations, we included VideoMol–^[^ ^14^ ^]^ a multimodal vision‐based drug representation model–as a unified baseline across all three tasks.

The results presented in Table 2 provide a comprehensive comparison of our method against various existing approaches across three distinct link prediction tasks: DMI (Drug–miRNA Interaction), DDI (Drug–Drug Interaction), and DTI (Drug–Target Interaction).

Table 2 presents a comparative evaluation of our proposed method, which integrates 1D fingerprints, 2D molecular graph convolutions, and 3D molecular visual representations, against several state‐of‐the‐art models across DMI, DDI, and DTI tasks. The results demonstrate that our feature fusion approach achieves the best performance in all three tasks, significantly outperforming existing methods in terms of ROC‐AUC, AUPR, Accuracy, Precision, and F1‐score.

For the DTI task, our method again achieves the best results, with a ROC‐AUC of 0.9761 and an AUPR of 0.9839, surpassing DrugBank, which achieves 0.9729 and 0.9685, respectively. The F1‐score (0.9661) is also the highest, demonstrating that leveraging multi‐scale molecular features provides richer information for drug‐target interaction prediction.

In the DDI task, our fusion approach significantly outperforms all baselines, achieving the highest ROC‐AUC (0.9812), AUPR (0.9651), and F1‐score (0.9648). Notably, compared to DSN‐DDI and MASMDDI, which rely on textual or structural features and achieve ROC‐AUCs of 0.9564 and 0.9565, respectively, our method improves by more than 2.5%, indicating that combining 1D, 2D, and 3D molecular representations enhances the ability to model drug‐drug interactions.

For the DMI task, our method achieves the highest ROC‐AUC (0.9858) and AUPR (0.9857), surpassing GAM‐MDR, the second‐best model, by 1.62% and 0.80%, respectively. The Accuracy (0.9393) and F1‐score (0.9403) also demonstrate the superiority of our multi‐view molecular representation in capturing drug‐microRNA interactions. The improvement over traditional molecular descriptors and learning‐based methods highlights the importance of integrating multi‐dimensional features.

In conclusion, our method consistently outperforms existing approaches across all three tasks, demonstrating its robustness and effectiveness in drug‐target interaction prediction. These results underscore the ability of our approach to capture complex patterns in drug‐related interactions, positioning it as a promising model for advancing drug discovery and interaction prediction research.

To contextualize these results, we compared MolVisGNN with VideoMol, a recent vision‐based framework that models drug molecules using rotating 3D molecular videos and transformer‐based encoding. While VideoMol achieves strong performance in small‐scale property prediction tasks, MolVisGNN offers distinct advantages in stereochemical sensitivity, multimodal integration, and task‐level generalization. Specifically, MolVisGNN employs six spatially fixed 3D views grouped into three orientation‐based pairs and processed through a direction‐aware convolutional encoder, enabling the capture of stereocenter orientation, ring conformation, and substituent positioning–features critical for distinguishing isomers with differing biological activities. In addition, MolVisGNN integrates 1D molecular fingerprints, 2D graph‐based topologies, and 3D visual features through a self‐controlled gated residual network (SCRN), dynamically weighting each modality without reliance on handcrafted clustering or surrogate supervision, thereby enhancing transparency, flexibility, and end‐to‐end learning efficiency. Furthermore, MolVisGNN demonstrates broader applicability by excelling in three biologically complex interaction prediction tasks–drug–target, drug–drug, and drug–microRNA–highlighting its capacity for interpretable and generalizable modeling across diverse molecular types and interaction mechanisms. From a system design perspective, MolVisGNN is computationally efficient, requiring no large‐scale pretraining and only six static 3D images per molecule, in contrast to VideoMol's high‐resolution video rendering and resource‐intensive pretraining. These advantages are comprehensively demonstrated in the supplementary materials, and a notable strength of MolVisGNN is that it achieves this leading position with substantially fewer parameters: 1 660 742 compared to 22 050 664 for VideoMol.

### 2.6 Ablation Experiments

We conducted ablation experiments on three benchmark datasets (DTI, DDI, and DMI) to evaluate the contribution of individual components in MolVisGNN. Specifically, MolVisGNN_1D + 2D_ removes the 3D molecular visual information, while MolVisGNN_splicing_ replaces the self‐controlled gated residual network (SCRN) with simple feature concatenation. As shown in **Table** **3**. Across all datasets, the complete MolVisGNN consistently achieved the best performance in ROC‐AUC, AUPR, accuracy, precision, and F1‐score. Removing the 3D component led to noticeable drops in structural discrimination, particularly for tasks with higher stereochemical complexity (e.g., DTI and DMI). Replacing SCRN with simple splicing caused further performance degradation, highlighting the importance of dynamic modality weighting for effective multimodal integration. These results demonstrate that both the 3D molecular view encoding and the SCRN module are critical for the model's predictive accuracy and generalization capability.

**Table 3:** Ablation experiments were performed on three datasets. MolVisGNN_1D + 2D_ represents the result of removing 3D information, and MolVisGNN_splicing_ represents the result of replacing SCRN with simple splicing.

Method	ROC‐AUC	AUPR	Accuracy	Precision	F1‐score
**DTI**					
**MolVisGNN**	**0.9761**	**0.9839**	**0.9660**	**0.9634**	**0.9661**
MolVisGNN_1D + 2D_	0.9657	0.9682	0.9393	0.9125	0.9412
MolVisGNN_splicing_	0.9394	0.9025	0.9180	0.8768	0.9222
**DDI**					
**MolVisGNN**	**0.9812**	**0.9651**	**0.9645**	**0.9560**	**0.9648**
MolVisGNN_1D + 2D_	0.9626	0.9524	0.9122	0.8765	0.9162
MolVisGNN_splicing_	0.9070	0.8896	0.8377	0.8038	0.8463
**DMI**					
**MolVisGNN**	**0.9858**	**0.9857**	**0.9393**	**0.9245**	**0.9403**
MolVisGNN_1D + 2D_	0.9397	0.9174	0.8710	0.8451	0.8756
MolVisGNN_splicing_	0.8662	0.8670	0.8221	0.8160	0.8239

### 2.7 Generalization to Novel Drug Scaffolds and Orphan Targets

**Figure 3:**
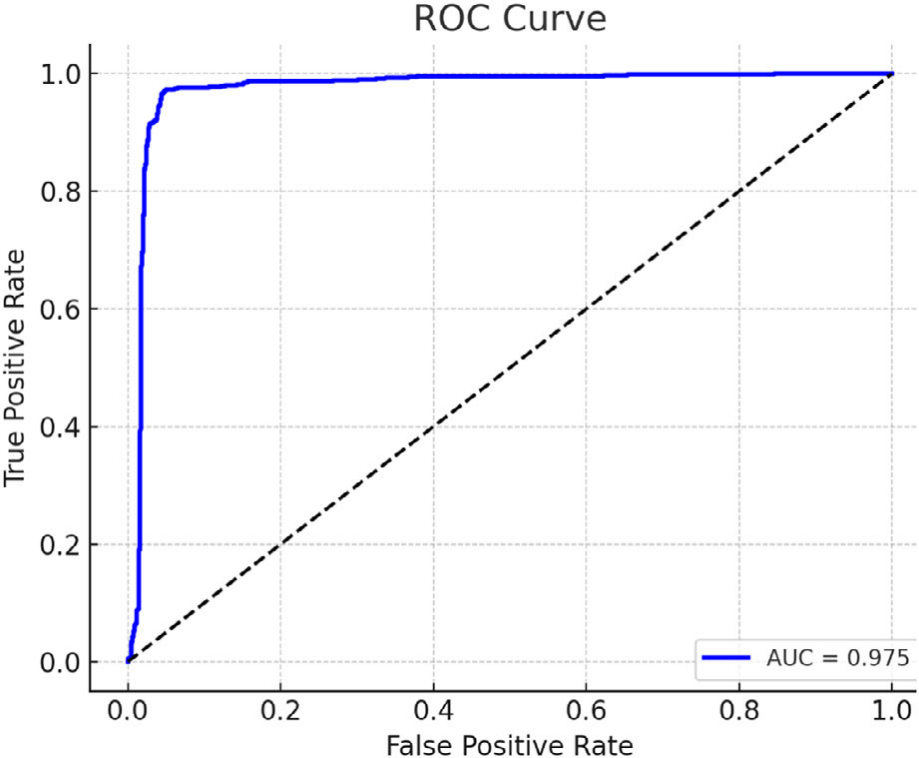
ROC for scaffold‐split evaluation.

To further assess the model's ability to generalize to novel chemical structures, we conducted a scaffold‐split experiment on the DMI dataset, where the training and test sets were partitioned based on Bemis–Murcko scaffolds to ensure no scaffold overlap. This setting simulates a more challenging and realistic discovery scenario, in which the model must make predictions for compounds with previously unseen core structures. Under this condition, our model achieved an AUC of 0.9750 and an AUPR of 0.9440, demonstrating that it can maintain high predictive accuracy even when confronted with entirely new scaffolds. The ROC–AUC curve is presented in **Figure** **3**. These results highlight the robustness of the proposed approach in handling scaffold‐level novelty, a key requirement for practical drug discovery applications.

In addition, we conducted an orphan target evaluation to assess the model's ability to make predictions for targets with no associated interactions in the training data. Specifically, in the DTI training set, all edges related to the drug Brivanib were removed while keeping the corresponding node in the graph. After model training, we constructed a balanced test set for Brivanib with a positive‐to‐negative ratio of 1:1. From the predictions on this set, we extracted the top five ranked examples based on predicted scores and presented them in **Table** **4**. Remarkably, all top‐ranked predictions corresponded to positive labels, demonstrating the model's strong capability to identify true interactions even under the stringent orphan target setting.

**Table 4:** Top‐5 predictions for Brivanib in the orphan target evaluation. All entries are positive cases.

UniProt ID	Protein name	Label
O60331	Phosphatidylinositol 4‐phosphate 5‐kinase type‐1 gamma (PIP5K1C)	Positive
Q14004	Serine/threonine‐protein kinase NEK9 (NEK9)	Positive
O14936	Mitogen‐activated protein kinase kinase kinase 7 (MAP3K7/TAK1)	Positive
Q96BR1	Serine/threonine‐protein kinase WNK1 (WNK1)	Positive
P11802	Cyclin‐dependent kinase 4 (CDK4)	Positive

### 2.8 Interpretable Experiments

In the current field of drug discovery, model interpretability is also an important part.^[^ ^31^ ^]^ We conducted various interpretability experiments on our model. Our fusion module adaptively assigns importance weights to three types of drug representations: 1D molecular fingerprints, 2D molecular graph convolutional representations, and 3D molecular spatial visual representations. To intuitively assess the contribution of each modality, we visualized the learned attention weights across three representative tasks–drug–target interaction (DTI), drug–drug interaction (DDI), and drug–microRNA interaction (DMI)–using nested donut charts (Figure S1, Supporting Information). The results reveal that 3D spatial information contributes substantially across all tasks: 39.8% in DTI (second only to fingerprints), 48.8% in DDI (dominant), and 32.3% in DMI (exceeding 1D fingerprints). These findings underscore the indispensable role of spatial conformation, often overlooked in traditional 1D or 2D encodings, in capturing interaction‐specific geometric cues.

**Figure 4:**
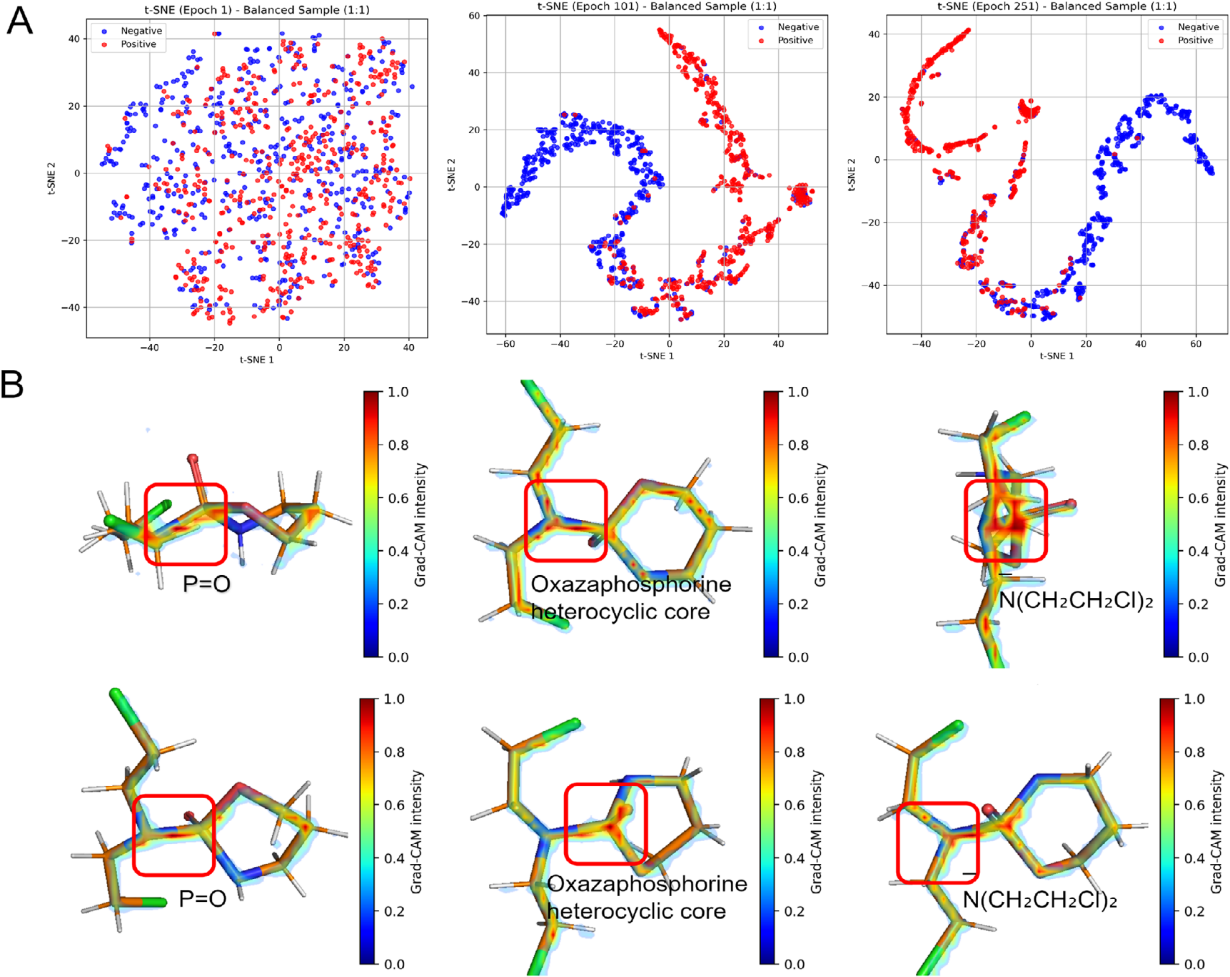
Visualization of learned representations, and spatial attention. A) t‐SNE visualization of the fused representation at epochs 1, 101, and 251, sampled with a 1:1 positive–negative ratio. With training progression, class separability improves significantly. B) Grad‐CAM visualization of the Cyclophosphamide molecule from six orthogonal views. Warmer colors (red/yellow) indicate stronger attention from the model, highlighting active regions such as ring structures and side chains critical to prediction.

To further explore the discriminative power of the learned representations, we applied t‐SNE to the fused feature vectors at different training stages (**Figure** **4**B). At epoch 1, positive and negative samples are inseparable in the latent space. However, as training proceeds, the t‐SNE plots exhibit clear class separation–evident by epoch 101 and strongly consolidated by epoch 251. These results suggest that the model progressively refines its internal representation, guided by multi‐perspective input, to better distinguish interaction‐relevant features. Importantly, this separation is achieved even under a balanced sampling setting (1:1 positive to negative), further affirming the robustness of the fused representation.

To interpret the spatial specificity of the model, we employed Gradient‐weighted Class Activation Mapping (Grad‐CAM)^[^ ^32^ ^]^ on the 3D molecular visual inputs (Figure 4C), with Cyclophosphamide as a case study. Six orthogonal molecular views highlight attention distributions, where heat intensity reflects the degree of model focus. High‐activation zones (red/yellow) localize around aromatic rings, chiral centers, and pharmacophoric substructures–regions typically associated with molecular recognition and activity.

Cyclophosphamide is a prototypical nitrogen mustard alkylating agent containing several key chemical moieties closely associated with its pharmacological activity. First, the phosphoramide group (P = O) features a phosphorus atom double‐bonded to oxygen and bound to both oxygen and nitrogen atoms, forming the molecule's primary electrophilic center. Second, the oxazaphosphorine ring, a five‐membered heterocycle composed of oxygen, nitrogen, and phosphorus, provides a specific spatial arrangement and pharmacophoric geometry. Finally, the chloroethyl side chains (–CH_2_CH_2_Cl) attached to nitrogen atoms constitute the direct alkylation sites, enabling covalent bonding with DNA bases and inducing crosslinking.

In the Grad‐CAM heatmaps, the red‐boxed regions across six orthogonal projection views consistently correspond to these functional groups. In the top‐left and bottom‐left views, the highlighted areas encompass the P = O bond and its adjacent oxygen and nitrogen atoms, reflecting the model's focus on the reactive center and its stereoelectronic effects. In the top‐middle and bottom‐middle views, the red boxes fully enclose the oxazaphosphorine ring, indicating recognition of its role in conformational stability and molecular recognition. In the top‐right and bottom‐right views, the high‐activation regions concentrate on the chloroethyl side chains and C–Cl bonds, showing attention to the alkylation reaction sites. These heatmaps illustrate the model's ability to selectively attend to structurally informative sites rather than uniformly over the molecular surface, aligning closely with established structure–activity relationships recognized by medicinal chemists. This demonstrates that the model not only captures the 3D geometry of key pharmacophores but also identifies functional groups and chemical features critical to biological activity, thereby providing chemically and biologically meaningful interpretability.

Together, the evolving t‐SNE clusters and Grad‐CAM heatmaps demonstrate that 3D molecular spatial visual representations provide complementary, biologically grounded information. Their integration not only improves predictive performance but also enhances model transparency in the context of drug–biomolecule interactions.

### 2.9 Case Study

To further evaluate the model's predictive capability, we selected two representative miRNAs (namely hsa‐let‐7c and hsa‐miR‐15a‐5p) and retrieved all associated drug–miRNA pairs from the test set. As shown in **Table** **5**, both miRNAs contain a mix of positive and negative interactions, with each having at least five associated samples. For both cases, the prediction scores for positive interactions were consistently high (all above 0.70), whereas the scores for negative interactions were markedly lower (below 0.30). All predictions were correct, demonstrating the model's strong discriminative ability in distinguishing true from false associations. These results highlight the robustness of the proposed approach in capturing biologically relevant miRNA–drug interaction patterns.

**Table 5:** Prediction scores, labels, and correctness for two selected miRNAs with mixed positive and negative cases.

Drug name	Score	Label	Predicted correctly
*hsa‐let‐7c*			
Tamoxifen	0.9478	Positive	Yes
Gemcitabine	0.9447	Positive	Yes
Vincristine	0.8423	Positive	Yes
Erlotinib	0.7073	Positive	Yes
Cabozantinib	0.0463	Negative	Yes
*hsa‐miR‐15a‐5p*			
Imatinib	0.9959	Positive	Yes
Docetaxel	0.9506	Positive	Yes
Etoposide	0.9339	Positive	Yes
Carboplatin	0.8720	Positive	Yes
Daunorubicin	0.7567	Positive	Yes
Praziquantel	0.2755	Negative	Yes

To further evaluate the practical applicability of our framework, we conducted a case study using Imatinib, a first‐line tyrosine kinase inhibitor approved for chronic myeloid leukemia (CML) and gastrointestinal stromal tumors (GIST). Its mechanism of action involves selective inhibition of oncogenic kinases such as BCR‐ABL, c‐KIT, and PDGFRA,^[^ ^33^ ^]^ thereby halting the proliferation of malignant cells.

We implemented a leave‐one‐out strategy by removing all nodes and edges associated with Imatinib from the training graph, simulating a cold‐start scenario in which the model has no prior exposure to the drug. During inference, the model was tasked with ranking candidate associations between Imatinib and microRNAs, other drugs, or protein targets.

Across all three prediction tasks, the model exhibited strong generalization ability. In the drug–microRNA interaction (DMI) task, 7 out of the top 20 microRNAs ranked by predicted scores were confirmed as true interactions. For the drug–drug interaction (DDI) task, 7 of the top 20 predicted drug partners were also previously known. Most notably, in the drug–target interaction (DTI) task, 18 of the top 20 ranked protein targets were experimentally validated Imatinib targets. These results suggest that our model can recover biologically meaningful relationships even under cold‐start conditions.

To probe the biological relevance of the predicted interactions, we analyzed potential regulatory links between the top‐ranked microRNAs and predicted protein targets. Given that microRNAs typically regulate protein expression through mRNA degradation or translational repression, such pathways may indirectly reflect compound mechanism‐of‐action. We selected the top five predicted protein targets and examined whether they were modulated by the highest‐ranking microRNAs in the same experiment.

**Figure 5:**
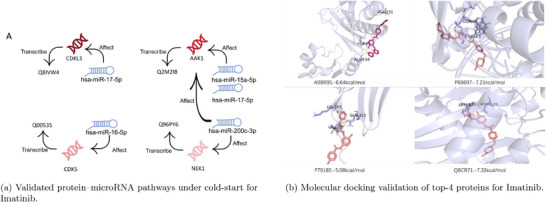
A) Validated protein–microRNA regulatory pathways predicted under the cold‐start setting for Imatinib. B) Molecular docking validation of top‐ranked proteins associated with Imatinib, selected from over 500 000 candidates.

We queried TarBase,^[^ ^34^ ^]^ a curated database of experimentally supported microRNA–gene interactions, to validate these indirect connections. As shown in **Figure** **5**A, we identified four representative regulatory cascades: hsa‐miR‐17‐5p connects to protein Q8IVW4 via CDKL3; hsa‐miR‐15a‐5p, hsa‐miR‐17‐5p, and hsa‐miR‐200c‐3p converge on Q2M218 through AAK1;^[^ ^35^ ^]^ hsa‐miR‐200c‐3p is linked to Q96PY6 via NEK1;^[^ ^36^ ^]^ and hsa‐miR‐16‐5p regulates Q00535 through CDK5.^[^ ^37^ ^]^ These findings suggest that the model captures layered biological interactions, not just direct binding events.

To assess the model's capability in de novo target discovery, we retrieved approximately 500 000 protein sequences from UniProt,^[^ ^38^ ^]^ excluding all known Imatinib‐related targets. Among the top predictions, we selected four high‐confidence candidates–Q8CR71, P79185, P68697, and A9BR95–for docking validation.

Q8CR71 encodes polyphosphate kinase from *Staphylococcus epidermidis*, an enzyme involved in energy metabolism and bacterial virulence,^[^ ^39^ ^]^ and a promising antimicrobial target.^[^ ^40^ ^]^ P79185 corresponds to the CD4 receptor in *Macaca fascicularis*, with high homology to human CD4, a critical immune co‐receptor and HIV entry factor.^[^ ^38^ ^]^ P68697 encodes Vaccinia virus DNA topoisomerase 1B, essential for relaxing supercoiled DNA during replication.^[^ ^41^ ^]^ A9BR95 is an uncharacterized protein from *Staphylococcus aureus* lacking current functional annotation,^[^ ^38^ ^]^ representing a potentially novel interaction candidate.

Molecular docking confirmed favorable binding affinities between Imatinib and all four proteins (Figure 5B), with predicted energies of −7.33 kcal/mol (Q8CR71), −5.08 kcal/mol (P79185), −7.21 kcal/mol (P68697), and −6.64 kcal/mol (A9BR95). These findings validate the model's utility in prioritizing novel, structurally compatible drug–target pairs, even when no training information about the compound is available.

Taken together, this case study demonstrates the capacity of our multimodal framework to identify direct and indirect drug–biomolecule interactions in data‐sparse settings. By combining 3D molecular spatial visual representations with complementary molecular features, the model supports generalizable and mechanistically interpretable predictions relevant to drug discovery and repositioning.

To evaluate the model's performance on non‐kinase inhibitors, we identified Thiodigalactoside, a non‐kinase inhibitor, in the DTI dataset as a case study. We ranked all prediction scores in the test set that were associated with Thiodigalactoside. Among the top ten ranked predictions, eight of the highest‐scoring entries corresponded to positive labels, as shown in **Table** **6**. This result further demonstrates the model's ability to prioritize true interactions for non‐kinase inhibitors.

**Table 6:** Top‐10 ranked predictions for Thiodigalactoside in the DTI test dataset, including prediction correctness.

Target_node	Score	Label	Predicted correctly
O00750	0.99995899	Positive	Yes
P00519	0.99995744	Positive	Yes
O95931	0.99993145	Positive	Yes
O43283	0.99989951	Positive	Yes
O95819	0.99987924	Positive	Yes
O43293	0.99987316	Positive	Yes
O15164	0.99985039	Positive	Yes
P11309	0.99927431	Negative	No
P16234	0.96791083	Negative	No
O00214	0.95742255	Negative	No

### 2.10 Evaluation on the LD_50_ Acute‐Toxicity Regression Task

We evaluated MolVisGNN on the LD_50_ dataset compiled by Zhu et al.,^[^ ^42^ ^]^ which aggregates experimental rat oral LD_50_ measurements from public sources (e.g., RTECS, ChemIDPlus), selecting the most conservative (lowest) value per compound. This benchmark comprises 7385 small‐molecule compounds with LD_50_ values spanning several orders of magnitude (mg/kg). On this acute‐toxicity regression task, MolVisGNN achieved a mean squared error (MSE) of 0.7348, a root mean squared error (RMSE) of 0.8572, a mean absolute error (MAE) of 0.6754 mg/kg, and a median absolute error (MedAE) of 0.5732 mg/kg, with an *R* ^2^ score of 0.2570 and an explained variance score (EVS) of 0.2695. These results indicate that MolVisGNN delivers consistent and competitive performance, effectively capturing the complex structure–toxicity relationships in small molecules.

In the LD_50_ regression task, 1,2,3,4‐Tetrachlorodibenzo‐*p*‐dioxin, the compound with the largest prediction deviation (AE = 5.4402), possesses a Murcko scaffold that appears only three times in the dataset, indicating extreme rarity. This scarcity likely hindered the model from learning reliable structure–toxicity associations, resulting in poor generalization. In contrast, Chlorpyrifos, the compound with the smallest deviation (AE = 0.00093), is built on a scaffold occurring 14 times in the dataset, enabling the model to capture its structural features more effectively and produce highly accurate predictions. This comparison highlights the influence of scaffold frequency on predictive performance, with rare scaffolds posing greater challenges for model generalization (**Table** **7**).

## 3 Discussion

Compared to traditional 1D or 2D drug representations, the proposed MolVisGNN uniquely incorporates a 3D molecular spatial visual modality, which captures fine‐grained geometrical and stereochemical cues that are often flattened or obscured in other descriptors. Empirically, this modality consistently outperforms classical fingerprints such as Topological Torsion, MACCS, and Morgan in drug–microRNA (DMI) and drug–drug interaction (DDI) tasks, while its slightly lower performance in drug–target interaction (DTI) prediction compared to Morgan fingerprints likely reflects the latter's close alignment with substructure‐based binding motifs. This highlights both the novelty and the complementary nature of our approach relative to established baselines.

To exploit multimodal complementarities, we designed a self‐controlled gate residual network (SCRN) to adaptively integrate 1D molecular fingerprints, 2D molecular graph embeddings, and 3D spatial visual features. This adaptive fusion consistently yields state‐of‐the‐art performance across DTI, DDI, and DMI tasks, demonstrating the advantage of dynamically weighting modalities according to task‐specific relevance. Furthermore, Grad‐CAM visualizations reveal that the model attends to chemically meaningful substructures–such as aromatic rings, functional branches, and scaffold cores–mirroring expert chemical intuition and reinforcing interpretability.

Our framework also performs well under challenging generalization settings. Under cold‐start conditions, using Imatinib as a withheld compound, the model's top predictions included microRNAs and protein targets embedded in plausible regulatory cascades, further supported by large‐scale screening of ∼500 000 UniProt proteins and docking validation of four novel targets with favorable binding affinities. In addition, scaffold‐based splitting ensured that training and test sets contained distinct chemical backbones, where MolVisGNN still achieved strong predictive performance. We further conducted orphan‐target experiments by removing all training interactions for Brivanib while retaining its node, and the model successfully recovered true associations in the top predictions. Finally, ablation studies confirmed the indispensability of both the 3D molecular modality and the SCRN fusion module, as removing either led to substantial performance degradation.

While promising, the present study is still limited in experimental scale, and more extensive benchmarking would further strengthen the conclusions. Moreover, wet‐lab validation will be an essential next step to confirm the biological and clinical relevance of our predictions.

## 4 Conclusion

In this study, we proposed a deep learning framework that integrates 3D molecular spatial visual representations with 1D molecular fingerprints and 2D graph‐based structures using a Self‐Controlled Gate Residual Network (SCRN) to predict drug–biomolecule associations. The model utilizes a 3D direction‐aware convolutional autoencoder to capture spatial geometry and stereochemical features that are often overlooked in traditional descriptors. Extensive experiments on drug–target, drug–drug, and drug–microRNA interaction tasks demonstrated that the fusion‐based framework consistently outperforms state‐of‐the‐art baselines in predictive accuracy and generalization. Interpretability analyses using Grad‐CAM revealed that the model attends to pharmacologically relevant substructures such as aromatic rings and functional side chains, confirming its ability to learn chemically meaningful representations. In a cold‐start case study with Imatinib, the model successfully recovered known targets and microRNA associations without prior exposure, and further uncovered multi‐step regulatory cascades supported by curated databases. Molecular docking validated four previously unrecognized but structurally plausible protein targets, illustrating the model's capability in novel target discovery. Together, these findings highlight the effectiveness of incorporating 3D spatial information into molecular modeling and demonstrate the potential of SCRN‐based multimodal integration in interpretable, generalizable, and application‐oriented drug discovery.

## 5 Experimental Section

### Datasets

To comprehensively evaluate the contribution of 3D molecular spatial visual representations, MolVisGNN was assessed on three representative tasks: drug–microRNA interaction (DMI) prediction, drug–drug interaction (DDI) prediction, and drug–target interaction (DTI) prediction. These tasks represent critical aspects of pharmacological modeling. MicroRNAs are key post‐transcriptional regulators implicated in drug response;^[^ ^43^ ^]^ drug–drug interactions may alter therapeutic efficacy or induce adverse effects; and protein targets serve as the primary mediators of drug action.

For the DMI task, 9732 drug–microRNA interaction pairs were obtained from the ncRNADrug database,^[^ ^44^ ^]^ involving 216 drugs and 2199 distinct microRNAs. The DDI task included 185 730 drug–drug interaction pairs across 1677 drugs from the DDInter database.^[^ ^45^ ^]^ The DTI task encompassed 46 495 drug–target interaction pairs from the Therapeutics Data Commons (TDC),^[^ ^46^ ^]^ covering 176 drugs and 503 protein targets. In all settings, unobserved or unannotated associations were treated as negative samples for training and evaluation.

To represent the non‐drug entities, we applied biologically informed encoding strategies tailored to each task. For microRNAs, sequences were retrieved from miRBase^[^ ^47^ ^]^ and encoded using k‐mer segmentation, which decomposes nucleotide chains into overlapping subsequences of length *k* to capture local sequence motifs and implicit secondary structure features. For proteins, ESM‐2 was used,^[^ ^48^ ^]^ a transformer‐based language model pretrained on large‐scale protein corpora, to extract evolutionary and structurally contextualized embeddings.

By integrating k‐mer‐derived microRNA features and ESM‐2‐based protein representations, we ensured that both interaction partners in DMI and DTI tasks were modeled using biologically meaningful and information‐rich descriptors. This consistent multimodal entity representation across tasks forms a robust foundation for learning drug–biomolecule interaction patterns.

As shown in **Table** **8**, the scaffold diversity varies considerably across the three benchmark datasets. The DTI dataset contains 176 molecules with 145 unique Murcko scaffolds, yielding a diversity ratio of 0.824 and a Shannon entropy of 4.827, indicating a relatively broad coverage of chemical space. In contrast, the DDI dataset includes 1677 molecules but only 992 unique scaffolds, resulting in a lower diversity ratio of 0.592 despite the highest Shannon entropy (6.096), reflecting a large dataset with several highly populated scaffold clusters. The DMI dataset, with 216 molecules and 170 unique scaffolds, exhibits a high diversity ratio (0.787) and moderate entropy (4.926), suggesting a balanced scaffold distribution. These differences highlight the varying degrees of chemical space coverage across tasks, which may influence model generalizability and predictive robustness.

**Table 7:** Comparison of the compounds with the largest and smallest prediction deviations in the LD_50_ regression task, including scaffold frequency and SMILES representation.

Compound name	AE	Scaffold frequency	SMILES
1,2,3,4‐Tetrachlorodibenzo‐*p*‐dioxin	5.4402	3	Clc1c(Cl)c2c(c(c1Cl)Cl)Oc3c2c(cc(c3)Cl)Cl
Chlorpyrifos	0.00093	14	CCOP(=S)(OCC)Oc1nc(Cl)c(Cl)cc1Cl

**Table 8:** Scaffold diversity statistics for the three benchmark datasets. The “Unique Murcko Scaffolds” column indicates the number of distinct Bemis–Murcko scaffolds after deduplication. Scaffold diversity is calculated as the ratio of unique scaffolds to valid molecules, and Shannon entropy quantifies the diversity of the scaffold distribution.

Task	Total molecules	Unique Murcko scaffolds	Scaffold diversity (Unique/Valid)	Shannon Entropy
DTI	176	145	0.824	4.827
DDI	1677	992	0.592	6.096
DMI	216	170	0.787	4.926

### Initialization of 1D and 2D Drug Representations

To construct initial molecular encodings, we first extract 1D and 2D features from distinct structural modalities. For 1D features, we employ classical molecular fingerprints, which convert the presence of predefined substructures, functional groups, and topological fragments into high‐dimensional binary vectors through rule‐based encoding schemes.

For 2D representations, we utilize the Weave Module–a graph convolutional architecture that simultaneously models atom‐level and bond‐level features within molecular graphs. This enables the capture of local interaction motifs and broader structural patterns crucial for molecular recognition.

The resulting fingerprint‐ and graph‐derived encodings are denoted as *x*_1_ and *x*_2_, respectively, and serve as input modalities for multimodal fusion.

### Drug 3D Molecular Spatial Visual Information Learning

To effectively capture the spatial organization and stereochemical complexity of drug molecules, we first converted SMILES strings into 3D structures in SDF format and rendered each molecule into six orthogonal views–front, right, back, bottom, top, and left–using PyMOL. These views preserve essential geometric features such as atomic positions, bond angles, and chiral centers, which are critical for inferring molecular function and interaction potential.

We propose a 3D direction‐aware convolutional autoencoder that integrates global and local spatial features of molecular structures. The model consists of two complementary components: (1) a global 3D convolutional encoder that extracts global backbone features–atomic connectivity, molecular shape, and functional group distribution–from six rendered views; and (2) a grouped direction‐aware convolution that focuses on fine‐grained local cues such as substituent orientation, ring conformation, and chirality. These substructural variations are often responsible for distinguishing structural isomers and stereoisomers, which may exhibit different pharmacological properties despite overall backbone similarities.

Each drug molecule is represented as a tensor $y_{i}\in R^{4\times 6\times H\times W}$, where the first dimension (4) corresponds to the four color channels used in rendering, the second dimension (6) corresponds to six orthogonal views of the 3D molecular structure, and *H* × *W* denotes the spatial resolution of each view. A stack of *n* 3D convolutional layers encodes this tensor to produce a global embedding:

(1) $$h_{k}=W_{k}*h_{k-1}+b_{k},\;h_{0}=y_{i}$$

(2) $$v=h_{n}$$

where * denotes 3D convolution, *W*_*k*_ and *b*_*k*_ are the trainable weights and biases of the *k*‐th layer, and *v* is the final embedding.

A decoder consisting of transposed 3D convolutional layers reconstructs the original input tensor:

(3) $$h_{l}^{\prime }=W_{d_{l}}⊛h_{l-1}^{\prime }+b_{d_{l}},\;h_{0}^{\prime }=x_{3}$$

(4) $${\hat{y}}_{i}=h_{m}^{\prime }$$

where ⊛ denotes transposed 3D convolution, $W_{d_{l}}$ and $b_{d_{l}}$ are the weights and biases of the *l*‐th decoder layer, and *x*_3_ is the seed tensor from the latent space.

The reconstruction loss is defined as

(5) $$L_{\mathrm{recon}}=\frac{1}{N}\sum_{i=1}^{N}{\left\|y_{i}-{\hat{y}}_{i}\right\|}_{2}^{2},$$

where *N* is the number of training samples.

This global representation captures shape complementarity, connectivity, and functional group layout.

To enrich the representation with local spatial asymmetries that differentiate isomers, we group the six view‐specific features into three directional pairs:

(6) $$g_{1}=\left[V_{\mathrm{front}},V_{\mathrm{right}}\right],\;g_{2}=\left[V_{\mathrm{back}},V_{\mathrm{bottom}}\right],\;g_{3}=\left[V_{\mathrm{top}},V_{\mathrm{left}}\right]$$

Each group *g*_*i*_ is processed through an independent direction‐aware 3D convolutional block:

(7) $$a_{1}={\mathrm{Conv}}_{\mathrm{fr}}\left(g_{1}\right),\;a_{2}={\mathrm{Conv}}_{\mathrm{bb}}\left(g_{2}\right),\;a_{3}={\mathrm{Conv}}_{\mathrm{tl}}\left(g_{3}\right)$$

This operation allows the model to capture pharmacologically meaningful spatial variations, such as substituent orientation, ring conformation, and chirality. The resulting feature maps are concatenated along the depth axis to produce the final directionally encoded 3D representation:

(8) $$x_{3}=\mathrm{Concat}\left(a_{1},a_{2},a_{3}\right)$$

**Figure 6:**
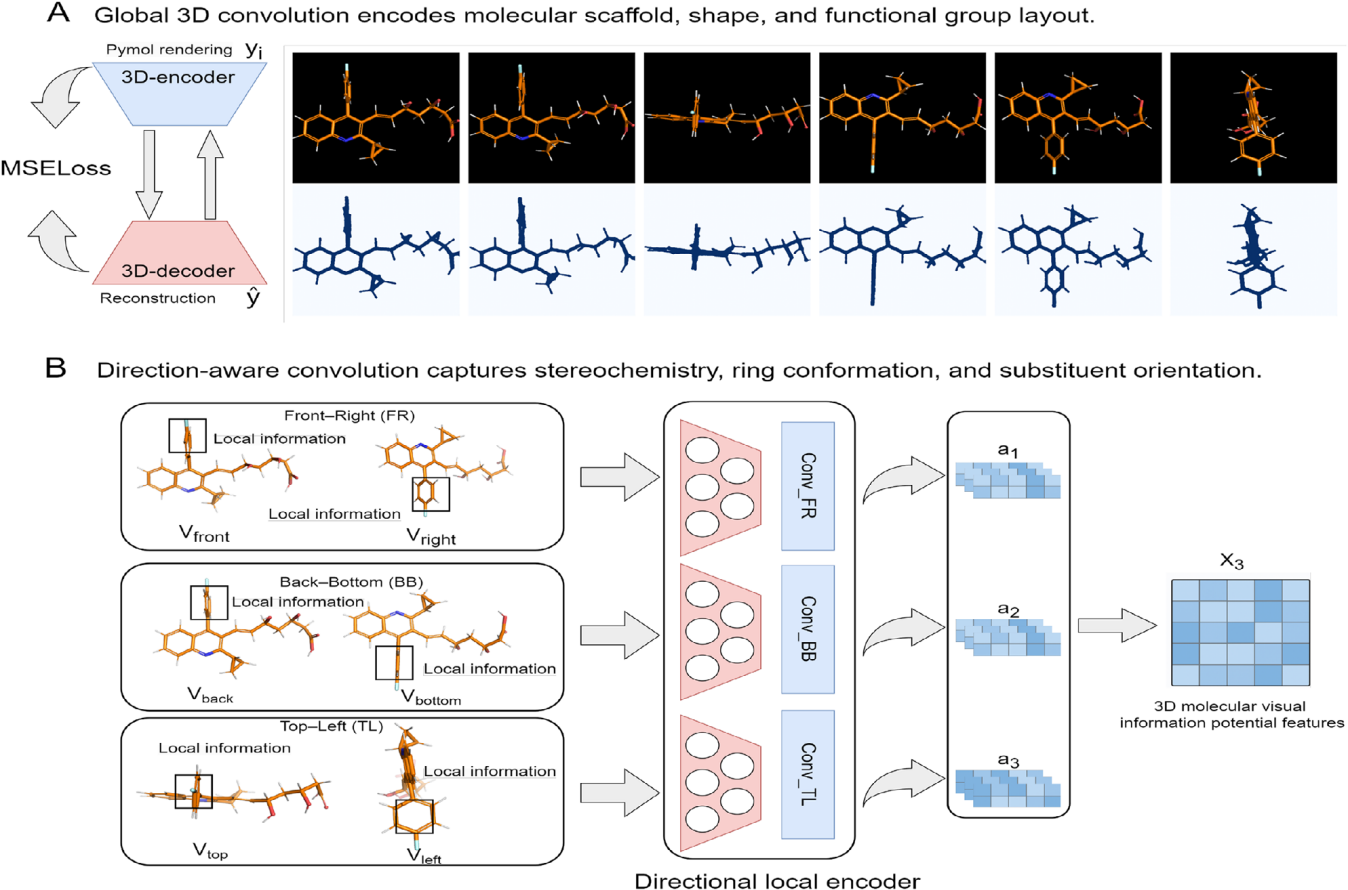
Schematic of the 3D direction‐aware convolutional autoencoder framework for extracting molecular spatial representations. (A) The global convolution module receives six orthogonal PyMOL‐rendered views of each molecule and encodes global structural features including the molecular scaffold, bonding patterns, and spatial conformation. Reconstruction is performed via a decoder trained with mean squared error (MSE) loss. (B) The direction‐aware convolutional module captures local spatial variations by dividing the six views into three directional pairs: front–right (FR), back–bottom (BB), and top–left (TL). Each pair is processed through an independent 3D convolutional path to extract orientation‐specific features such as substituent geometry, ring conformations, and chiral centers. The resulting directional features (*a*_1_, *a*_2_, *a*_3_) are concatenated along the depth axis to form a fused 3D representation *x*_3_ for downstream tasks.

Through this architecture **Figure** **6**, the encoder learns representations that jointly encode global scaffold topology and local stereochemical distinctions. Such features are essential for accurately modeling isomer‐sensitive binding affinity, enabling applications in virtual screening, activity prediction, and drug–target interaction modeling.

### 5.1 Multi‐Perspective Drug Representation Learning

To effectively integrate heterogeneous drug representations, we propose a modality‐aware fusion framework that combines three complementary views: 1D molecular fingerprints (*x*_1_), 2D graph‐based molecular convolutional representations (*x*_2_), and 3D molecular visual representations (*x*_3_). Each modality encapsulates distinct and biologically relevant aspects of molecular structure. Molecular fingerprints provide a compact encoding of functional groups and pharmacophoric substructures, the 2D graph captures atom connectivity and chemical bonding patterns, and the 3D view encodes spatial conformation, which is essential for modeling stereochemistry, molecular docking, and target binding affinity.

Each modality is first mapped into a shared latent space through a learnable linear transformation:

(9) $$\begin{array}{cc} z_{1} & =W_{1}x_{1}+b_{1} \end{array}$$

(10) $$\begin{array}{cc} z_{2} & =W_{2}x_{2}+b_{2} \end{array}$$

(11) $$\begin{array}{cc} z_{3} & =W_{3}x_{3}+b_{3} \end{array}$$

This transformation aligns the semantic and numerical scales of features across modalities, enabling effective downstream integration. From a biological perspective, this alignment allows the model to unify structural motifs, topological connectivity, and spatial geometry into a common embedding space that reflects the functional landscape of drug molecules.

To adaptively assess the relative importance of each modality, the transformed embeddings are passed through a softmax function to generate modality‐specific attention weights:

(12) $$w_{i}=\frac{e^{z_{i}}}{\sum_{j=1}^{3}e^{z_{j}}},\;i=1,2,3$$

This weighting mechanism allows the model to dynamically emphasize the most informative modality for each compound. For example, in large and rigid molecules with complex stereochemistry, the 3D modality may dominate due to its ability to represent spatial asymmetry and molecular symmetry. In contrast, for small, flexible molecules or those with linear scaffolds, 1D fingerprints or 2D graph features may offer stronger predictive signals due to their capacity to capture substructure presence and bonding patterns.

The final fused representation is computed via a weighted concatenation of the original input features:

(13) $$X=[w_{1}x_{1};\;w_{2}x_{2};\;w_{3}x_{3}]$$

This produces a comprehensive feature vector that incorporates information from multiple structural levels and biochemical dimensions.

To further extract nonlinear and higher‐order interactions among modalities, the fused representation *X* is passed through a 1D residual convolutional encoder comprising two sequential convolutional layers. The first convolution captures localized dependencies among feature dimensions:

(14) $$h_{1}=f_{1}\left(W_{1}*X+b_{1}\right)$$

The second convolution extracts more abstract and semantically rich patterns:

(15) $$h_{2}=f_{2}\left(W_{2}*h_{1}+b_{2}\right)$$

Finally, a residual connection integrates the initial input with the learned high‐level features:

(16) $$y=h_{2}+X$$

This design enhances information preservation and facilitates gradient propagation. The resulting representation *y* captures both modality‐specific characteristics and their biologically meaningful interactions, thereby improving the model's ability to support downstream tasks such as drug–target interaction prediction, drug–RNA interaction profiling, and structure‐based drug repurposing.

### GCN for Downstream Task

The learned fused representation *y*, which integrates 1D, 2D, and 3D molecular visual representations of drug molecules, serves as input to the downstream interaction prediction module. While the fusion representation captures rich intra‐molecular information across multiple structural levels, it does not explicitly model the topological relationships between drugs and targets. To address this, we adopt a graph convolutional network (GCN) framework, which enables the integration of molecular representations with known drug–target associations by propagating feature signals across a relational graph. This allows the model to learn from both feature‐level similarities and the connectivity structure of the drug–target interaction network.

We denote drug representations as $X\in R^{n\times f_{d}}$ and target features as $T\in R^{m\times f_{t}}$. These are concatenated to form the initial node feature matrix $H^{\left(0\right)}\in R^{(n+m)\times f}$, where *f* = *f*_*d*_ + *f*_*t*_. The adjacency matrix $A\in R^{\left(n+m)\times (n+m\right)}$ is constructed based on known drug–target interaction pairs. Specifically, for drug–drug interaction (DDI) prediction, *A* encodes pairwise drug associations; for drug–miRNA (DMI) prediction, *A* is defined as a bipartite graph linking drugs and miRNAs; and for drug–target interaction (DTI) prediction, *A* connects drugs with protein targets.

To propagate information over the graph and capture complex topological dependencies, we apply a graph convolutional network (GCN). The propagation from layer *l* to layer *l* + 1 follows:

(17) $$H^{\left(l+1\right)}=\sigma \left({\tilde{D}}^{-\frac{1}{2}}\tilde{A}{\tilde{D}}^{-\frac{1}{2}}H^{\left(l\right)}W^{\left(l\right)}\right),$$

where $\tilde{A}=A+I$ is the adjacency matrix with added self‐connections, $\tilde{D}$ is its degree matrix, *H* ^(*l*)^ is the node representation at the *l*‐th layer, and *W* ^(*l*)^ denotes the layer‐specific trainable weights. The non‐linear activation function σ plays a crucial role in model training. In our implementation, we use

(18) σ(·)=LeakyReLU(·).

which alleviates the dying ReLU problem by allowing a small, non‐zero gradient when the unit is not active.

After *L* layers of propagation, the final node representation matrix *H* ^(*L*)^ is obtained, from which we extract the updated drug features *X*_final_ and target features *T*_final_. These representations are then used to predict drug potential relationship discovery task.

We employ a sigmoid activation to compute the interaction probability matrix $\hat{Y}\in R^{n\times m}$, where ${\hat{Y}}_{ij}$ denotes the predicted probability of interaction between the *i*‐th drug and *j*‐th target. The model is trained by minimizing the binary cross‐entropy loss:

(19) $$L=-\frac{1}{n\times m}\sum_{i=1}^{n}\sum_{j=1}^{m}\left[Y_{ij}\log\left({\hat{Y}}_{ij}\right)+\left(1-Y_{ij}\right)\log\left(1-{\hat{Y}}_{ij}\right)\right],$$

where *Y* ∈ {0, 1}^*n* × *m*^ is the ground truth interaction matrix.

## Conflict of Interest

The authors declare no conflict of interest.

## Data Availability

The data that support the findings of this study are available from the corresponding author upon reasonable request.
